# Comparative Analysis of Intrathecal Bupivacaine With Fentanyl Versus Intrathecal Bupivacaine With Midazolam in Lower Abdominal and Lower Limb Surgeries

**DOI:** 10.7759/cureus.68908

**Published:** 2024-09-07

**Authors:** Jayant Bhatia, Chhaya Suryawanshi

**Affiliations:** 1 Anaesthesiology, Dr. D.Y. Patil Medical College, Hospital and Research Centre, Dr. D.Y. Patil Vidyapeeth (Deemed to be University), Pune, IND

**Keywords:** analgesia, intrathecal fentanyl, intrathecal midazolam, sedation, sensory, spinal anesthesia

## Abstract

Many different adjuvants are added intrathecally along with local anaesthetics to prolong intraoperative and postoperative analgesia. Hence, this study aimed to compare intrathecal bupivacaine with fentanyl and bupivacaine with midazolam in lower abdominal and lower limb surgeries. Following permission from the Hospital Ethical Committee (Research Protocol No.: IESC/PGS/2022/143), the study was conducted on a sample of 60 patients, divided into two groups, Group F and Group M, with 30 patients each, representing the American Society of Anaesthesiologist's (ASA) grades I and II. The patients were between the ages of 18 and 60 and featured both males and females, scheduled to undergo elective surgical procedures on the lower abdomen and lower limbs via spinal anaesthesia. Group “F”: 3 ml of 0.5% hyperbaric bupivacaine hydrochloride with 0.5 ml (25 mcg) of fentanyl (preservative-free) intrathecally. Group “M”: 3 ml of 0.5% hyperbaric bupivacaine hydrochloride with 0.5 ml (2.5 mg) of midazolam (preservative-free) intrathecally. The primary aim was to study the onset of motor and sensory block and duration of analgesia with the addition of midazolam or fentanyl to 0.5% heavy bupivacaine in the sub-arachnoid block. A secondary aim was to evaluate the quality of anaesthesia and postoperative analgesia, determine the haemodynamic stability in the intraoperative and postoperative period in the two study groups, and observe any adverse effects of study drugs. Group F exhibited a substantially longer duration for both sensory (211.5 vs. 154.4 min) and motor blockade (269.8 vs. 214.6 min) compared to Group M, as well as a rapid onset time for both sensory (2.6 vs. 3.3 min) and motor blockade (3.1 vs. 3.9 min). Also, Group F had a significantly longer duration of effective analgesia compared to Group M (266 ± 15.9 vs. 197.6 ± 13.7 minutes). The addition of 0.5 ml (2.5 mg) Midazolam with 0.5% hyperbaric Bupivacaine intrathecally prolonged the duration of anaesthesia and postoperative analgesia; however, fentanyl 0.5 ml (25 mcg) has a more prolonged duration of intraoperative and postoperative analgesia.

## Introduction

The subarachnoid block is the most commonly used regional anaesthesia technique for lower abdomen or infra-umbilical surgeries. Nowadays, spinal anaesthesia is preferred because of its quick onset, superior action, reduced risk of infection, lower failure rates, and cost-effectiveness [1].

Various adjuvants can be used with neuraxial anaesthetics to expedite the onset, improve the effectiveness, and expand the range of subarachnoid block. The drugs that can be used are opioids like fentanyl and morphine, vasoconstrictor agents like adrenaline, gamma-aminobutyric acid (GABA) receptor agonists like midazolam, and alpha-2-adrenoceptor agonists like clonidine. When an adjuvant is combined with a local anaesthetic medication, it enhances the impact and extends the duration of intra-operative and post-operative pain relief. This aids in decreasing the requirement for analgesics after surgery and the dosage of bupivacaine. However, the utilisation of these adjuvants comes with inherent hazards because they can also cause unwanted side effects such as respiratory depression, itching, urinary retention, somnolence, nausea, and gastrointestinal distress. Bupivacaine hydrochloride is a predominantly and economically efficient local anaesthetic employed in spinal anaesthesia. Nevertheless, managing pain after surgery is a significant challenge because of the brief duration of action of spinal anaesthesia when incorporating just local anaesthetics. Consequently, prompt administration of analgesics is necessary during the postoperative period [2].

Intrathecal opioids are commonly used as adjuvants. They attach to opioid receptors located in laminae I and II of the dorsal horn, both before and after the synapse. Activation of the µ and δ opioid receptors leads to the opening of potassium (K^+^) channels through G protein mediation, whereas activation of the κ opioid receptor results in the closure of calcium channels. These events result in a decrease in the amounts of calcium within the cells, which in turn reduces the release of excitatory neurotransmitters, specifically glutamate and leads to a decrease in pain relief [3].

Fentanyl and morphine are the predominant opioids in application. Fentanyl is a type of opioid agonist that specifically targets µ-opioid receptors. It is commonly administered through the intrathecal route to enhance the anesthesia’s quality and duration. It exhibits a higher lipid solubility than morphine, resulting in faster elimination from the cerebral fluid and a quick initial therapeutic effect. It offers a stable hemodynamic state, high-quality pain relief during surgery, and long-lasting pain relief after surgery with few adverse effects [4].

Midazolam is a short-acting, potent, water-soluble benzodiazepine. It has been used for potentiating the analgesic effects of neuroaxial blockade. The potential mechanism behind midazolam’s antinociceptive effect is through the stimulation of the benzodiazepine/gamma amino butyric acid-A receptor expressed in the lamina II of the spinal cord’s dorsal horn [5].

Therefore, the aim of this study was to compare intrathecal bupivacaine and fentanyl with bupivacaine and midazolam in lower abdominal and lower limb surgeries.

## Materials and methods

Institute Ethics Committee clearance was obtained before the start of the study (Research Protocol No.: IESC/PGS/2022/143). The study was conducted on a sample of 60 patients, both males and females, scheduled to undergo elective surgical procedures on the lower abdomen and/or lower limbs via spinal anesthesia.

Patients eligible for inclusion were ASA Grade I or II, aged between 18 and 60, scheduled to undergo lower abdominal or lower limb surgeries under spinal anesthesia. They needed to be hemodynamically stable, not on cardiac-related medications, and able to provide written informed consent.

Exclusion criteria included patients unwilling to participate, those with ASA physical status III or higher, individuals outside the 18 - 60 age range, and those undergoing emergency procedures. Additionally, patients with uncontrolled systemic disorders, spine deformities, local skin interactions, bleeding or coagulation disorders, or known allergies to the study drugs were excluded.

The materials required for the procedure included a standard anesthesia machine (Boyle’s apparatus), a 20 G intravenous (IV) cannula, IV fluids (both crystalloids and colloids), and monitoring equipment such as a pulse oximeter, electrocardiogram (ECG) monitor, and noninvasive blood pressure apparatus. A sterile spinal tray, a 26 G Quincke’s spinal needle, disposable syringes, and drugs for spinal anesthesia (0.5% hyperbaric bupivacaine hydrochloride 4 ml ampoule, inj. fentanyl 50 mcg/mL ampoule, and inj. midazolam 5 mg/mL ampoule) were also required. Additionally, drugs and equipment necessary for resuscitation were on hand.

The day before surgery, a preoperative visit was conducted to take a comprehensive medical history and record any complaints. A thorough assessment of the cardiovascular, respiratory, and central nervous systems was performed, and routine laboratory investigations were completed, including a haemogram, liver function tests, renal function tests, serum electrolytes, urine routine, bleeding time and clotting time (BT-CT), and PT/INR. Patients were instructed to abstain from eating or drinking for at least 6 - 8 hours prior to surgery. Additionally, they were given a detailed explanation of the procedure, and written informed consent was obtained from them after providing the necessary information.

In the operating room, standard anesthesia monitors were connected, and baseline parameters such as preoperative pulse, noninvasive blood pressure, ECG, and oxygen saturation were recorded. Peripheral venous access was established, and IV fluids were started, with preloading of the patient with 10 - 15 ml/kg body weight of Ringer’s lactate over 10 - 20 minutes.

A subarachnoid block was administered in the sitting position under strict aseptic precautions. After preparing and draping the lumbar area, a 26 G Quincke’s spinal needle was inserted into the L3-L4 intervertebral space. Cerebrospinal fluid (CSF) flow was confirmed, and depending on the group, the respective drugs were injected intrathecally: Group F received a mixture of 3 ml of 0.5% hyperbaric bupivacaine hydrochloride and 0.5 ml (25 mcg) of fentanyl, whereas Group M received 3 ml of 0.5% hyperbaric bupivacaine hydrochloride and 0.5 ml (2.5 mg) of preservative-free midazolam. Following the injection, the patient was positioned supine and adjusted to achieve a sensory block up to the T6 segment. Pulse and blood pressure were monitored at predetermined intervals.

Sensory block was assessed by performing a pinprick test along the left mid-clavicular line until the block reached the T6 level, at which point surgical incision was permitted. To evaluate the onset of blockade, the following readings were recorded: T0 was the time of subarachnoid block; T1 was the time of onset of sensory block, indicated by the loss of pinprick sensation; and T2 was the time of onset of motor block, determined by the inability to lift the extended leg.

Motor blockade was evaluated using the Bromage scale (Table 1) [6].

**Table 1:** Bromage scale

Grade	Criteria	Degree of block
1	Free movement of legs and feet	Nil
2	Just able to flex knees with free movement of feet	Partial (33%)
3	Unable to flex knees, but free movement of feet	Almost complete (66%)
4	No movement	Complete (100%)

During the intra-operative period, patients were closely monitored for pulse rate, respiratory rate, SpO_2_, blood pressure, and blood loss, with whole blood administered as necessary to replace any significant loss. Side effects such as nausea, drowsiness, constipation, sedation, hypotension, and respiratory depression were recorded and managed with appropriate medications. IV infusion was maintained with Ringer’s lactate, 5% dextrose, and normal saline. The quality of the blockade was assessed using the Ramsay Sedation Score (Table 2) [7].

**Table 2:** Ramsay Sedation score

Sedation score	Response
1	Anxious and agitated or restless or both
2	Cooperative, oriented, and tranquil
3	Responds to commands only
4	Brisk response to a light glabellar tap or loud auditory stimulus
5	Sluggish response to light glabellar tap or loud auditory stimulus
6	No response to stimulus

**Figure 1:**
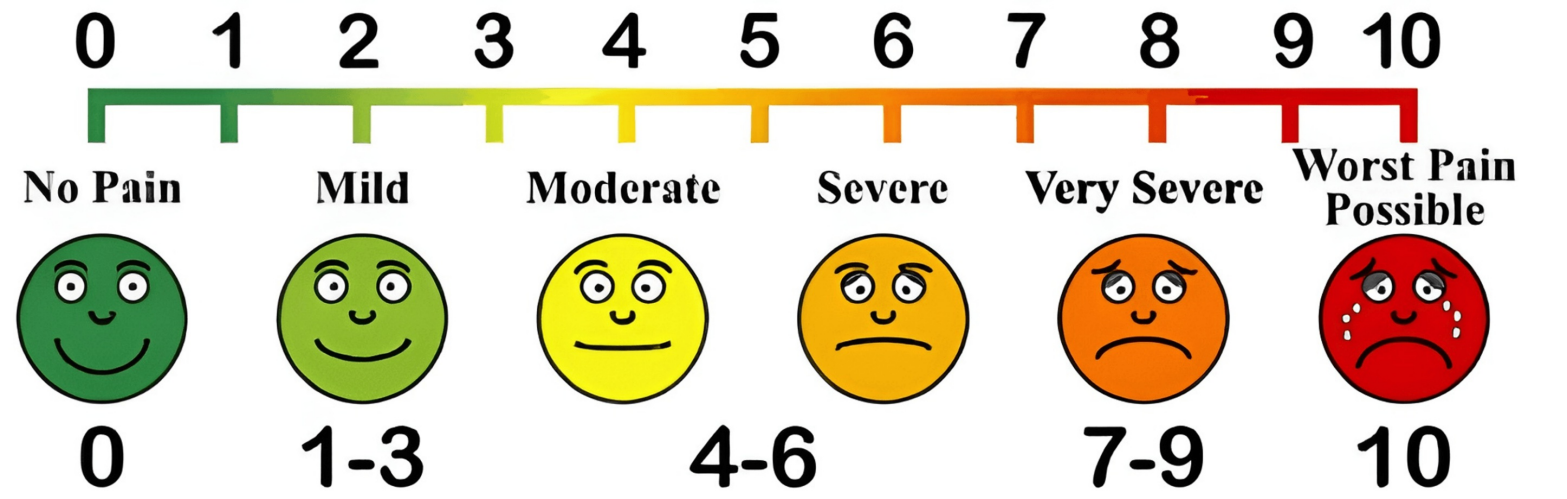
Visual Analogue Scale

After surgery, patients were transferred to the post-operative care unit for standard monitoring before being moved to their respective wards. The efficacy of post-operative analgesia was evaluated using the Visual Analogue Scale (VAS) (Figure 1).

Subsequent rescue analgesics (inj. tramadol 50 mg) was given if the patient had a VAS of 5 or more than 5. The duration of effective analgesia was assessed from the moment of subarachnoid block administration until the patient requested the initial rescue analgesic. T3 refers to the time at which rescue analgesia was administered.

Statistical analysis

The collected data were processed into Microsoft Excel 2019 (Microsoft® Corp., Redmond, WA), and data analysis was conducted using Statistical Package for Social Sciences for Windows, Version 22 (IBM SPSS, Armonk, NY). The categorical variables were represented by the symbol “n,” whereas the continuous variables were displayed as the mean and standard deviation (SD). The distribution of categorical variables was compared using either the chi-square test or Fisher's exact test. Continuous variables were compared using a student t-test. A significance level of p-value < 0.05 was used to determine statistical significance.

## Results

Demographic profile

The majority of the patients in Group F belonged to the 31-40 and 51-60 age groups (30%). In Group M, the majority of patients belonged to the 31-40 age group; there was not a statistically significant difference in age distribution between the two groups (p > 0.05) (Table 3).

**Table 3:** Age-based frequency distribution

Age distribution	Group F (n = 30)	Group M (n = 30)
<30 year	6 (20%)	5 (16.7%)
31–40 years	9 (30%)	9 (30%)
41–50 years	6 (20%)	8 (26.7%)
51–60 years	9 (30%)	8 (26.7%)
Total	30 (100%)	30 (100%)
P-value	0.93	

Most of the patients in Group F (83.3%) and M (80%) were female. There was no statistically significant difference in gender distribution between the two groups (p > 0.05) (Table 4).

**Table 4:** Gender-based frequency distribution

Gender	Group F (n = 30)	Group M (n = 30)
Male	5 (16.7%)	6 (20%)
Female	25 (83.3%)	24 (80%)
Total	30 (100%)	30 (100%)
P-value	0.73	

The majority of the Group F (66.7%) and Group M (73.3%) patients belonged to ASA class I, and the difference in frequency distribution was not statistically significant when chi-square test was applied (p > 0.05) (Table 5).

**Table 5:** ASA grading-based distribution

ASA	Group F (n = 30)	Group M (n = 30)
I	20 (66.7%)	22 (73.3%)
II	10 (33.3%)	8 (26.7%)
Total	30 (100%)	30 (100%)
P-value	0.57	

In Group F, the majority of patients had a faster onset of sensory block with a prolonged mean duration of sensory block, but in Group M, the majority of patients had slower onset of sensory block with a shorter mean duration of sensory block. Mean and SD were calculated. In Group F, mean onset of sensory block was 2.6 ± 0.5 minutes with a mean duration of sensory block of 211.5 ± 17.6 minutes, but in Group M, mean onset of sensory block was 3.3 ± 0.6 minutes with a mean duration of sensory block of 154.4 ± 15.4 minutes. The quantitative analysis of the mean onset and duration of sensory block was conducted among the groups, and a statistically significant difference was observed (p-value < 0.05) (Table 6).

**Table 6:** Comparison of onset and duration of sensory block

Parameters	Group F (n-30)	Group M (n-30)	P-value
Time of onset of sensory block (min)	2.6 ± 0.5	3.3 ± 0.6	<0.001
Duration of sensory block (min)	211.5 ± 17.6	154.4 ± 15.4	<0.001

In Group F, patients had a faster onset of motor block with a prolonged mean duration of motor block, but in Group M, patients had slower onset of motor block with a shorter mean duration of motor block. Mean and SD were calculated. In Group F, mean onset of motor block was 3.1 ± 0.78 minutes with a mean duration of motor block of 269.8 ± 18.4 minutes, but in Group M, mean onset of motor block was 3.9 ± 0.8 minutes with a mean duration of motor block for 214.6 ± 15.8 minutes. Quantitative analysis of the mean onset and duration of motor block was conducted among the groups, and a statistically significant difference was observed (p-value < 0.05) (Table 7).

**Table 7:** Comparison of onset and duration of motor block

Parameters	Group F (n-30)	Group M (n-30)	p-value
Time of onset of motor block (min)	3.1 ± 0.78	3.9 ± 0.8	<0.001
Duration of motor block (min)	269.8 ± 18.4	214.6 ± 15.8	<0.001

In Group F, the mean heart rate (beats per minute) at baseline, after subarachnoid block, at 1, 5, 10, 30, 60 minutes, and end of surgery was 71.2 ± 6.7, 59.1 ± 5.7, 65.8 ± 4.6, 68.2 ± 3.2, 69.2 ± 2.4, 71.2 ± 2.4, 73.2 ± 2.4, and 75.2 ± 2.4, respectively, whereas in Group M, the mean heart rate was 73.3 ± 3.1, 64.1 ± 5.5, 69.1 ± 10.9, 71.3 ± 4.6, 71.9 ± 4.1, 73.9 ± 4.1, 75.9 ± 4.1, and 77.9 ± 4.1, respectively. Mean heart rate was statistically significant after subarachnoid block, at 1, 5, 10, 30, 60 minutes, and at end of surgery (p < 0.05) (Table 8).

**Table 8:** Heart rate distribution in two groups at different intervals

Heart rate (BPM)	Group F (n-30)	Group M (n-30)	P-value
Baseline	71.2±6.7	73.3±3.1	0.12
Before drug	68.4±5.7	70.2±3	0.13
After subarachnoid block	59.1±5.7	64.1±5.5	<0.001
1 min	65.8±4.6	69.1±10.9	0.01
3 mins	65.3±5.4	70.5±4.1	0.13
5 mins	68.2±3.2	71.3±4.6	<0.001
10 mins	69.2±2.4	71.9±4.1	0.004
30 mins	71.2±2.4	73.9±4.1	0.003
1 hour	73.2±2.4	75.9±4.1	0.003
End of surgery	75.2±2.4	77.9±4.1	0.003

In Group F the mean systolic blood pressure (SBP) at baseline, after subarachnoid block, at 1, 5, 10, 30, 60 minutes, and end of surgery was 127.2 ± 3.9, 108.4 ± 11.9, 101.3 ± 7.9, 107.07 ± 3.1, 112.6 ± 3.5, 113.8 ± 4.5, 115.8 ± 4.6, and 117.8 ± 4.6, respectively, whereas in Group M, the mean SBP was 124 ± 8.1, 94.07 ± 3.6, 100.2 ± 3.7, 100.6 ± 6.8, 103.6 ± 7.2, 108.3 ± 8.9, 110.3 ± 8.9, and 112.3 ± 8.9, respectively. Mean SBP was statistically significant after subarachnoid block, at 1, 5, 10, 30, 60 minutes, and at end of surgery (p < 0.05) (Table 9).

**Table 9:** Systolic blood pressure distribution SBP: systolic blood pressure

SBP (mmHg)	Group F (n-30)	Group M (n-30)	P value
Baseline	127.2±3.9	124±8.1	0.06
Before drug	121.7±5.3	119.7±6.3	0.18
After subarachnoid block	108.4±11.9	94.07±3.6	<0.001
1 min	101.3±7.9	100.2±3.7	<0.001
3 mins	106.2±17.7	99.1±8.3	0.51
5 mins	107.07±3.1	100.6±6.8	0.053
10 mins	112.6±3.5	103.6±7.2	<0.001
30 mins	113.8±4.5	108.3±8.9	<0.001
1 hour	115.8±4.6	110.3±8.9	0.004
End of surgery	117.8±4.6	112.3±8.9	0.004

In Group F, the mean diastolic blood pressure (DBP) at baseline, after subarachnoid block, at 1, 5, 10, 30, 60 minutes, and end of surgery was 72.3 ± 6.7, 68.3 ± 5.9, 66.9 ± 5.7, 63.8 ± 4.4, 66.8 ± 4.4, 66.1 ± 2.7, 68.1 ± 2.8, and 70.1 ± 2.8, respectively, whereas in Group M, the mean DBP was 70.7 ± 4.4, 60.5 ± 3.2, 57.9 ± 2.3, 59.1 ± 3.4, 58.7 ± 2.8, 60.7 ± 3.1, 62.7 ± 3.1, and 64.7 ± 3.1 respectively. Mean DBP was statistically significant after subarachnoid block, at 1, 5, 10, 30, 60 minutes, and at end of surgery (p < 0.05) (Table 10).

**Table 10:** Diastolic blood pressure distribution DBP: diastolic blood pressure

DBP (mmHg)	Group F (n = 30)	Group M (n = 30)	P-value
Baseline	72.3±6.7	70.7±4.4	0.27
Before drug	68.07±5.1	66.5±1.5	0.12
After subarachnoid block	68.3±5.9	60.5±3.2	<0.001
1 min	66.9±5.7	57.9±2.3	<0.001
3 mins	66.8±4.7	58.6±2.1	<0.001
5 mins	63.8±4.4	59.1±3.4	<0.001
10 mins	66.8±4.4	58.7±2.8	<0.001
30 mins	66.1±2.7	60.7±3.1	<0.001
1 hour	68.1±2.8	62.7±3.1	<0.001
End of surgery	70.1±2.8	64.7±3.1	<0.001

In Group F, the mean arterial pressure (MAP) at baseline, after subarachnoid block, at 1, 5, 10, 30, 60 minutes, and end of surgery was 90.5 ± 4.8, 78.2 ± 1.9, 78.3 ± 3.8, 78.1 ± 3.5, 81.9 ± 3.4, 82.4 ± 1.9, 80.01 ± 3.1, and 82.01 ± 3.1, respectively, whereas in Group M, the mean MAP was 88.8 ± 1.7, 76.6 ± 3.7, 72.4 ± 2.9, 72.1 ± 2.7, 74.5 ± 3.1, 76.0 ± 4.4, 78.3 ± 2.8, and 80.3 ± 2.8, respectively. Mean MAP was statistically significant after subarachnoid block, at 1, 5, 10, 30, and 60 minutes, and at the end of surgery (p < 0.05) (Table 11).

**Table 11:** Mean arterial blood pressure distribution MAP: mean arterial pressure

MAP	Group F (n-30)	Group M (n-30)	P-value
Baseline	90.5±4.8	88.8±1.7	0.07
Before drug	85.4±1.7	83.9±3.9	0.07
After subarachnoid block	78.2±1.9	76.6±3.7	0.03
1 min	78.3±3.8	72.4±2.9	<0.001
3 mins	79.6±7.2	71.8±3.5	<0.001
5 mins	78.1±3.5	72.1±2.7	<0.001
10 mins	81.9±3.4	74.5±3.1	<0.001
30 mins	82.4±1.9	76.04±4.4	<0.001
1 hour	80.01±3.1	78.3±2.8	0.02
End of surgery	82.01±3.1	80.3±2.8	0.03

Mean sedation score was 2 ± 0, 2.5 ± 0.5, and 2.5 ± 0.5, respectively, in Group F, but, it was 2 ± 0, 2.7 ± 0.47, and 2.5 ± 0.5, respectively, in Group M. Group F exhibited a comparatively lower sedation score in comparison to Group M, and the difference was not found to be statistically significant (p > 0.05) (Table 12).

**Table 12:** Sedation Score comparison

Sedation score	Group F (n-30)	Group M (n-30)	p-value
Pre-operative	2±0	2±0	-
10 mins after Subarachnoid block	2.5±0.5	2.7±0.47	0.29
Post-operative	2.5±0.5	2.5±0.5	1.0

In our study, the duration of effective pain relief, which is the time it took for the first request for more pain relief, was substantially longer in Group F compared to Group M. The duration was 266 ± 15.9 minutes in Group F and 197.6 ± 13.7 minutes in Group M. Group F exhibited a comparatively prolonged effective analgesia in comparison to Group M, and the observed difference was determined to be statistically significant (p < 0.05) (Table 13).

**Table 13:** Time for rescue analgesia

Parameters	Group F (n = 30)	Group M (n = 30)
Time for rescue analgesic (mins)	266±15.9	197.6±13.7
P-value	<0.001	

Comparison between VAS Scores was done at 1 hour, 2 hours, 3 hours, and 4 hours post-surgery in both groups. Both groups reported no pain one hour after the surgery, as indicated by a VAS score of 0. Most patients in Group F showed lower VAS score within four hours following the operation. Conversely, individuals in Group M experienced discomfort and exhibited higher VAS scores. More precisely, 33.3% of the patients indicated a VAS score of 4 three hours following the surgery, whereas all participants reported a VAS score of 5 four hours after the procedure in Group M. This demonstrates that the comparison was statistically significant (p < 0.05) (Table 14).

**Table 14:** Comparison of Visual Analogue Scores between both groups

Group F (n-30)					Group M (n-30)			
VAS	1 hour	2 hour	3 hour	4 hour	1 hour	2 hour	3 hour	4 hour
0	30 (100%)	25 (83%)	19 (63.3%)	0	30 (100%)	19 (63%)	0	0
1	0	5 (16%)	8 (26.6%)	1 (3.33%)	0	6 (20%)	0	0
2	0	0	3 (10%)	9 (30%)	0	5 (16%)	1 (3.33%)	0
3	0	0	0	9 (30%)	0	0	8 (26.6%)	0
4	0	0	0	9 (30%)	0	0	10 (33.3%)	0
5	0	0	0	2 (6.6%)	0	0	11 (36.6%)	30 (100%)
Total	30 (100%)				30 (100%)			
P-value	<0.001							

In Group F, the majority of patients did not experience any side effects, except for one patient who had nausea/vomiting. However, in Group M, two patients had nausea/vomiting, and three patients experienced shivering. This comparison of side effects between two groups was statistically insignificant (p > 0.05) (Table 15).

**Table 15:** Side-effects in study groups

Side effects	Group F (n-30)	Group M (n-30)	p-value
Nausea/vomiting	1 (3.3%)	2 (6.6%)	0.16
Shivering	0	3 (10%)	
Nil	29 (96.7%)	25 (83.3%)	

## Discussion

The primary concern during and after surgery is typically pain. The presence of pain during and after surgery can have detrimental impacts on a patient's mental and physical well-being as well as their overall recovery process. Therefore, an anaesthesiologist must possess the expertise and pharmacological understanding to alleviate intraoperative and postoperative pain and to effectively manage pain in all circumstances. There is now widespread recognition that inadequate pain management and the accompanying stress response have significant physiological and psychological consequences. These consequences can lead to organ dysfunction and increase postoperative mortality and morbidity rates [8].

In spinal anaesthesia, 0.5% bupivacaine heavy is a frequently employed local anaesthesia because of its prolonged duration of action and its profound sensory and motor block. Bupivacaine "heavy" is a hyperbaric solution of bupivacaine hydrochloride denser than CSF. This property enables the anaesthetic to settle in the dependent regions of the subarachnoid space, thereby ensuring a controlled and predictable distribution of anaesthesia [9].

Using intrathecal adjuvants in combination with local anaesthetics has gained significant popularity in enhancing intra-operative and post-operative analgesia. Adjuvants to hyperbaric bupivacaine in spinal anaesthesia include a variety of medications. Recent research has demonstrated that the use of fentanyl and midazolam as adjuvants increases the period of sensory blockade and analgesic effects, intraoperatively and postoperatively [10].

Midazolam is a pharmacological agent that has a short duration of action and is used to induce sleep and reduce anxiety. It also has anticonvulsant and amnestic effects. It is classified as a benzodiazepine drug. This medication distinguishes itself from other drugs in its class by its prompt onset of effects and brief duration of action. Midazolam enhances the opening frequency of chloride channels by binding to the GABA-A-BZD-Cl complex, resulting in hyperpolarization of the cell membrane [11].

Fentanyl, a synthetic opioid, is a lipophilic agonist of the µ receptor. It has a rapid onset of action; unlike morphine, it is less likely to migrate at sufficiently high concentrations, which could cause delayed respiratory depression. It functions by attaching to opioid receptors located on the dorsal horn of the spinal cord. It might spread and function supraspinal [12].

The majority of patients in Group F were in the age range of 31 to 40 years and 51 to 60 years, whereas the majority in Group M were in the 31 to 40 age range. Female patients constituted the majority of Group F (83.3%) and Group M (80%). The majority of patients in Group F (66.7%) and Group M (73.3%) had ASA class I. There were no statistically significant differences in age, gender, and frequency distribution across the groups.

In our study comparing the addition of fentanyl and midazolam to hyperbaric bupivacaine for spinal anaesthesia, we found consistent results favouring fentanyl in terms of onset (2.6 vs. 3.3 minutes) and duration (211.5 vs. 154.4 minutes) of sensory block as well as onset (3.1 vs. 3.9 minutes) and duration (269.8 vs. 214.6 minutes) of motor block.

Gupta et al. [13] found that fentanyl significantly reduced the onset time and extended the duration of sensory (4.54 minutes and 227 minutes) and motor blocks (7.68 minutes and 192 minutes) compared to bupivacaine alone (sensory: 7.1 minutes and 196 minutes vs. motor: 8.51 minutes and 183.60 minutes). Meanwhile, midazolam (sensory: 4.04 minutes and 254 minutes vs. motor: 7.64 minutes and 201.20 minutes) also prolonged these side effects, such as bradycardia and hypotension, hence corroborating our study.

Suraj et al. [14] reported that fentanyl (Group F) had a faster onset of sensory block (144.5 ± 14.01 seconds) and longer duration (172.30 ± 5.08 minutes) compared to midazolam (Group M) (onset: 165.4 ± 11.61 seconds; duration: 147.30 ± 8.71 minutes). Similarly, in our study, Group F had a faster onset and longer duration of motor block compared to Group M.

Ambuj et al. [15] also demonstrated that fentanyl led to a faster onset (144.50 ± 14.01 seconds) and longer duration (172.30 ± 5.0 minutes) of sensory block compared to midazolam (onset: 165.40 ± 11.61 seconds; duration: 147.30 ± 8.7 minutes).

Katakam et al. [16] observed that while the motor block durations were similar between groups (fentanyl: 161.66 ± 15.58 minutes; midazolam: 165.12 ± 14.30 minutes), our study found fentanyl had a quicker onset (3.1 ± 0.78 minutes) and longer duration (269.8 ± 18.4 minutes) of motor block compared to midazolam (onset: 3.9 ± 0.8 minutes; duration: 214.6 ± 15.8 minutes).

Sawhney et al. [17] found a greater decrease in heart rate with fentanyl (22.3 ± 17.9 bpm) compared to midazolam (11.7 ± 12.7 bpm) and better postoperative pain control with lower VAS scores.

The study by Srikanth and Amrutha [18] and our study (266 vs. 197.6 minutes) both showed that fentanyl provides a longer duration of analgesia (264.32 ± 15.38 minutes) compared to midazolam (246.2 ± 14.3 minutes), with comparable haemodynamic stability and sedation.

Puja et al. [19] found fentanyl provided a longer duration of pain relief (293.16 ± 35 minutes) than midazolam (267.80 ± 32 minutes), with fewer side effects. This result is comparable with the results of our study (266 vs. 197.6 minutes).

Shweta et al. [20] reported fewer intraoperative adverse effects with fentanyl compared to midazolam, supporting our finding that fentanyl alone resulted in fewer side effects compared to midazolam.

Finally, Nayak et al. [21] found that fentanyl provided a longer duration of postoperative analgesia (367.73 minutes) compared to midazolam (254.9 minutes). These results aligned with our study’s results, which showed that fentanyl offers superior pain control and longer-lasting pain relief (266 minutes) in comparison to midazolam (197.6 minutes).

Limitations

The current investigation was constrained by a limited sample size. Furthermore, our investigation was carried out on patients classified as ASA-I and II, between the age range of 18 to 60 years. Additional research is needed to determine the efficacy of involved drugs in high-risk patients who are old or have reduced heart function. The VAS score is a subjective method utilized to assess the duration of analgesia and the timing of rescue analgesia.

## Conclusions

Intrathecal 0.5% hyperbaric bupivacaine with 0.5 ml (25 mcg) fentanyl resulted in faster onset of both sensory (2.6 vs. 3.3 mins) and motor (3.1 vs. 3.9 mins) block, with a longer duration of sensory (3.53 vs. 2.58 hours) and motor block (4.5 vs. 3.58 hours), as well as a longer duration of post-operative pain relief (4.4 vs. 3.28 hours) compared 0.5 ml (2.5 mg) midazolam as an adjuvant with 0.5% hyperbaric bupivacaine.

The addition of 0.5 ml (2.5 mg) midazolam with 0.5% hyperbaric bupivacaine intrathecally also prolonged the duration of anaesthesia and postoperative analgesia, but fentanyl 0.5 ml (25 mcg) had a more prolonged duration of intra-operative and post-operative analgesia. No major side effects were observed in both the study groups.
